# Myo-Inositol’s Role in Assisted Reproductive Technology: Evidence for Improving the Quality of Oocytes and Embryos in Patients With Polycystic Ovary Syndrome

**DOI:** 10.7759/cureus.8079

**Published:** 2020-05-12

**Authors:** Deepti Gupta, Safeera Khan, Muhammad Islam, Bilal Haider Malik, Ian H Rutkofsky

**Affiliations:** 1 Reproductive Medicine, Saint Mary's Hospital, Manchester, GBR; 2 Obstetrics and Gynecology, California Institute of Behavioral Neurosciences and Psychology, Fairfield, USA; 3 Internal Medicine, California Institute of Behavioral Neurosciences and Psychology, Fairfield, USA; 4 Pediatrics, California Institute of Behavioral Neurosciences and Psychology, Fairfield, USA; 5 Psychiatry, California Institute of Behavioral Neurosciences and Psychology, Fairfield, USA

**Keywords:** pcos, polycystic ovary syndrom, inositol, oocyte quality, embryo quality, ivf, art, assisted conception, invitrofertilisation

## Abstract

Polycystic ovary syndrome (PCOS) is one of the most common causes of subfertility, and it is characterized by hormonal dysregulation like insulin resistance. Various measures have been taken in the past to overcome this insulin resistance to improve fertility treatment outcomes. The current paper aims to review and compare the existing studies and literature to assess the impact of myo-inositol (MI) on oocyte and embryo quality in assisted reproductive technology (ARTs). We thoroughly searched the PubMed and Google Scholar databases by using the keywords “PCOS, polycystic ovarian syndrome, inositol, oocyte quality, embryo quality, assisted conception, ART, IVF, and in vitro fertilization.” Nine articles were finalized for review in this paper. Many of the reviewed studies have shown a trend toward the improvement of embryo quality in women with PCOS after MI supplementation; however, there is a lack of statistically significant evidence to support the use of MI in enhancing the quality of oocyte and/or embryo. Clear evidence regarding the role of MI in enhancing the quality of oocyte and embryo in PCOS is limited. A well-controlled, large, randomized controlled trial is required to definitively accept or refute its role.

## Introduction and background

Polycystic ovary syndrome (PCOS) is a common reproductive condition associated with chronic anovulation; it commonly manifests as oligomenorrhea, irregular menstrual cycle, and androgen excess, with typical ovarian ultrasound features [1]. It is the most prevalent cause of disorder of ovulation and subfertility in females and affects approximately 6-10% of childbearing women population [2]. Although its pathogenesis is poorly understood, the role of insulin in the pathogenesis of hyperandrogenemia in PCOS is central. Insulin resistance in association with luteinizing hormone (LH) increases the production of androgen in theca cells [3]. Therefore, treatment with an insulin-sensitizing agent like inositol, troglitazone, or metformin in women with PCOS may lead to the resumption of spontaneous ovulation [4-8].

Many recent studies have shifted interest toward the two main inositol stereoisomers out of the nine isomers of the inositol family, namely myo-inositol (MI) and D-chiro-inositol (DCI). This inositol complex acts as a second messenger of insulin signaling. Both of these isomers have insulin-like action and have therefore been claimed to improve various menstrual and hormonal parameters in PCOS. Studies on DCI has shown its ability to improve the chances of ovulation and reduction of androgen production in women with PCOS.

PCOS cannot be merely considered as a local ovarian dysfunction, but it is the expression of a complex functional alteration of the whole reproductive system [4,5]. Various randomized and nonrandomized cohort studies have shown that inositol complex (MI and DCI) improves menstrual irregularities and ovarian activity in women with PCOS [8,9]. The benefit of MI supplementation in infertility treatment is due to its ability to increase the intracellular calcium ion oscillation. It has been shown that the follicular fluid of oocytes containing a higher concentration of MI is of better quality in humans [9]. This has also been demonstrated in mouse germinal vesical oocytes by improved meiotic progression [10]. Additionally, it has been stated that the mechanism of inositol could prove beneficial in ways other than its action on the reduction of insulin resistance. For instance, it has been shown that MI is important for follicle-stimulating hormone (FSH) signaling and, therefore, for oocyte maturation and embryo development. The international consensus conference has stated that pretreatment with inositol(s) supplementation could improve oocyte quality and obstetric outcome for in vitro fertilization (IVF) patients [4,11].

In this paper, we aimed to review the role of inositol complex on oocyte and embryo quality in women with PCOS who had undergone various assisted reproductive technology (ART) treatments.

## Review

Methods

We systematically searched Pubmed and Google Scholar databases for relevant published articles to find studies assessing the effect of inositol complex supplementation and its effect on oocyte quality in IVF. Keywords we used for the search include “PCOS, polycystic ovarian syndrome, inositol(s), oocyte quality, embryo quality, in vitro fertilization, assisted conception, IVF, and ART.” There was no restriction regarding the time period in our search for the articles. A summary of the outcomes of the search is as follows: a) the keyword “polycystic ovarian syndrome” returned 16,263 peer-reviewed articles; b) “PCOS” yielded 14,659 peer-reviewed articles; c) 185 articles were returned for the combined keywords “PCOS and inositol”; d) 21 articles were found with combined keywords “PCOS, inositol, and in vitro fertilization”; e) when the combined keywords “PCOS, inositol, in vitro, and oocyte quality” were searched, 17 articles were listed. Nine articles were ultimately finalized for review. In this study, systematic review and meta-analysis of randomized controlled trials in which inositol supplementation was used in PCOS cases that underwent ART cycles are included, along with medical hypotheses, observational studies, and prospective trials.

Discussion and results

PCOS and Insulin Resistance

PCOS is a disorder characterized by insulin resistance and hyperinsulinemia. These features occur in both obese and nonobese women. Insulin resistance and hyperinsulinemia are considered to play an important role in the pathogenesis of hyperandrogenic production, ovulatory dysfunction, and various factors of metabolic syndrome in PCOS [2]. These are found in up to 75% of lean PCOS and around 95% of obese PCOS women [12]. The relationship between hyperinsulinemia and hyperandrogenism in polycystic ovarian disease has been identified by Burghen et al. [13]. In PCOS, insulin resistance leads to compensatory hyperinsulinemia. The reduced level of sex hormone-binding globulin and excess ovarian androgen production in PCOS women are the result of compensatory hyperinsulinemia. It plays a prominent role in the pathogenesis of various metabolic syndromes and anovulatory cycle [14,15]. Various actions have been recommended to overcome this insulin resistance as a first-line intervention, such as physical exercise, dietary and lifestyle modification, and insulin sensitizers. However, they are usually unable to overcome insulin resistance, and further interventions are often required [12,16,17].

Inadequate insulin action could be because of the deficiency of DCI. DCI is a component of inositol phosphoglycans (IPGs), which are the second messengers in the insulin pathway. MI, an insulin-sensitizing agent, helps in the restoration of ovulation and on oocyte meiotic maturation. Inositol affects the process of steroidogenesis and reduces the production of androgen from theca cell and decreases the serum concentration of testosterone [4,6,18,19].

Myo-Inositol and Its Role in Insulin Resistance

In 1850, Johann Joseph von Scherer discovered a new compound from a muscle cell and called it inositol, which was coined by combining various Greek words [20,21]. Inositol belongs to the vitamin B complex family. The chemical formula of inositol substance is C6H12O6OR (-CHOH)6. The food items that naturally contain the highest concentration of inositols are fruits, beans, corn, and nuts, indicating that the inositol is widely available in nature [22]. Nine stereoisomers of the inositol family are currently known, and MI is the most common isomer available; DCI is the second most common form. These isomers are formed by the epimerization of six hydroxyl groups of inositol, and MI and DCI are used in the treatment of PCOS as insulin-sensitizing agents [4,11,23-25]. Inositol was formerly known as “myometrial sugar,” although it is not a member of the carbohydrate family. Indeed, the direct involvement of the inositol molecule in insulin signaling has been proven in various studies.

**Figure 1:**
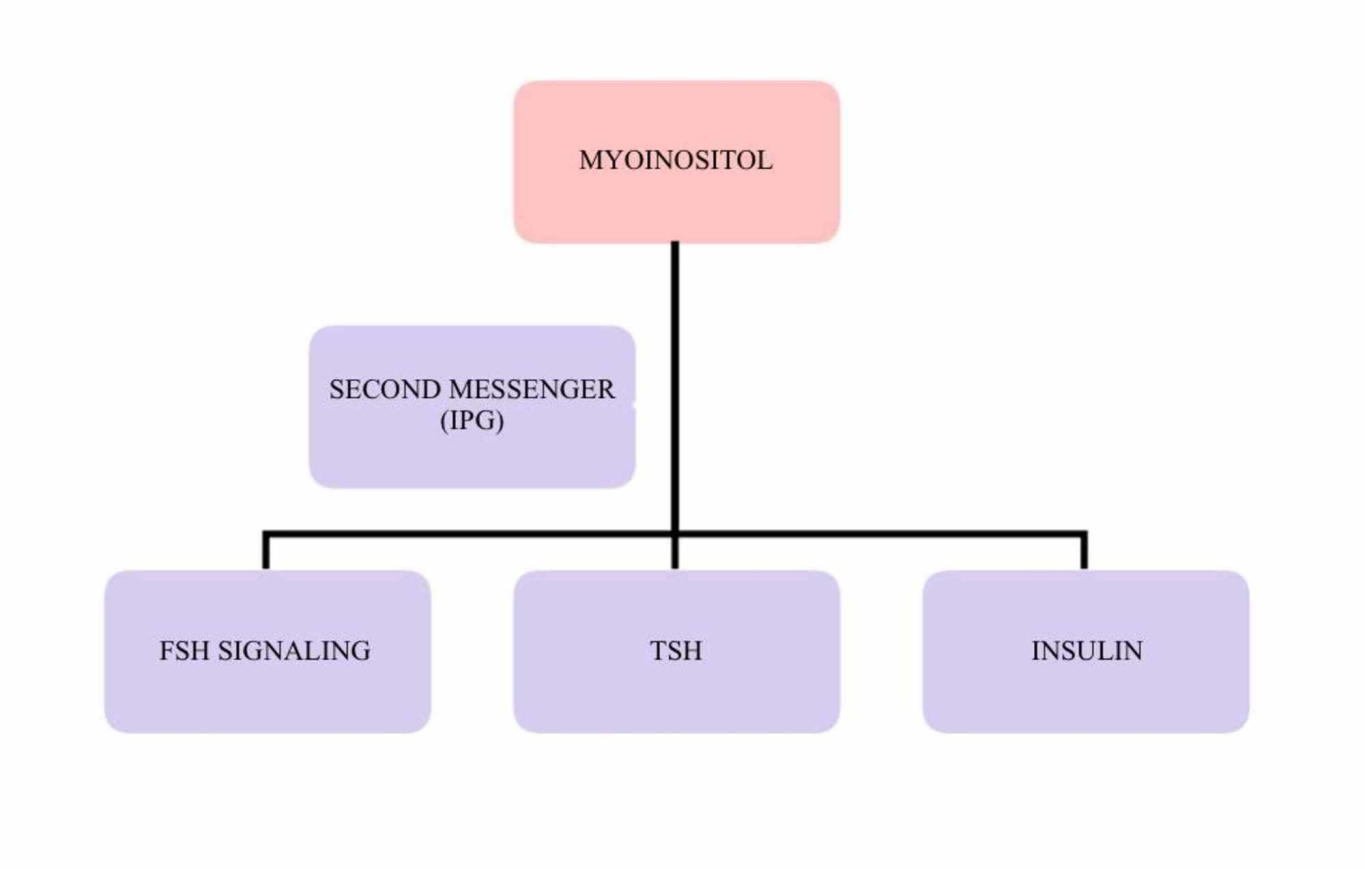
Role of myo-inositol as a second messenger IPG: inositol phosphoglycan; FSH: follicle-stimulating hormone; TSH: thyroid-stimulating hormone

Inositol is involved in regulating a multitude of hormonal signals and metabolic pathways in human beings [25,26]. Over the last few decades, various studies have emphasized the “insulin-sensitizing properties” of inositols. Both MI and DCI appear to be capable of activating the main enzymes involved in the metabolism and uptake of glucose [26-28]. MI is converted into DCI by the activity of the epimerase enzyme [29]. IPGs are the second messengers of insulin. MI and DCI are incorporated intracellularly into IPG, and these IPG mediators mediate some of the actions of the insulin. Phosphatidyl-myo-inositol is the phosphatidyl-insphosphate precursor, and its hydrolysis results in inositol triphosphate. This acts as a second messenger in the regulation of various hormone activities such as those of thyroid-stimulating hormone (TSH), FSH, and insulin. It also helps in improving their signals, as shown in Figure 1 [30,31].

It has been evident from various studies that defect in IPGs' second messenger results in insulin pathway impairment [32,33]. This is because IPGs play a role in the activation of enzymes that control the metabolism of glucose [34,35]. Insulin resistance in women with PCOS is due to the defect in either tissue availability or altered metabolism of IPGs' mediator or inositol. Various studies have shown that MI is capable of restoring spontaneous ovarian activity in women with PCOS and, therefore, fertility in many of these cases [25].

PCOS, Oocyte Quality, and Inositol

One of the main challenges in ART is the difficulty in obtaining good-quality oocyte and embryos. Various studies have been conducted to find out the factors that can predict IVF outcomes. The most common reason for unsuccessful IVF-embryo transfer procedures is insufficient oocyte and embryo quality. Other then this, several other reasons like social and environmental factors, aging, and other pathologies can also negatively affect the outcome of the procedure.

In women with PCOS, the oocytes retrieved are often found to be of poor quality [36]. Interestingly, many of these women with PCOS need ARTs to achieve pregnancy. However, over two-thirds of ART cycles result in adverse pregnancy outcomes. This is primarily attributed to the failure in fertilization due to poor oocyte quality [37]. Any treatment capable of improving the quality of the oocyte could, therefore, be considered as a “crowning achievement” for ART procedures. This is the reason why IVF techniques now focus mainly on getting a better quality of oocytes rather than higher numbers of eggs and embryos [38]. PCOS is one of the most common ovulatory disorders, and it is characterized by hormonal dysfunction. It is commonly seen that a higher proportion of women with PCOS have hyperinsulinemia and insulin resistance. Therefore, insulin sensitizers are used to counteract the above-mentioned hormonal signs due to the pathophysiological link between insulin resistance and PCOS aberrations.

MI has been found to be essential for the proper maturation of oocytes, and its higher concentration in human follicular fluid is considered as a marker of high oocyte quality [10,19]. Studies have shown that MI supplementation is positively correlated with the meiotic progression of germinal vesicle oocytes of the mouse by increasing intracellular Ca2+ oscillation [10]. It is also important to note that the ovary maintains normal sensitivity to insulin despite systematic insulin resistance [36]. In women with PCOS, an increase in insulin leads to the stimulation of ovarian epimerase activity. This increased epimerase activity results in a higher level of DCI and a lower level of MI in a follicular fluid, which is called “the ovarian paradox” [39]. As MI is involved in FSH signaling, its depletion in follicular fluid in PCOS could lead to impaired FSH signaling [18,40]. The studies included in this review are summarized in Table 1.

**Table 1:** Studies included in the review MI: myo-inositol; PCOS: polycystic ovary syndrome; IVF: in vitro fertilization; DCI: D-chiro-inositol; ICSI: intracytoplasmic sperm injection; ET: embryo transfer

No.	Journal	Author	Type of study	year	Aim of the study	Patient population (if applicable)	Conclusions
1	International Journal of Endocrinology	Lesoine B et al. [41]	Original article	2016	To find out if the MI + folic acid combination was able to improve the quality of oocyte, the ratio between follicles and retrieved oocytes, the fertilization rate, and embryo quality	29 patients with PCOS	PCOS women with MI supplementation showed better fertilization rates and improved embryo quality
2	European Review for Medical and Pharmacological Sciences	Ciotta et al. [42]	Original article	2011	To determine the effects of MI on oocytes quality in women with PCOS	34 patients with PCOS	A higher number of oocytes were retrieved in the MI group. The number of immature oocytes was also less in the inositol group
3	Hormone Molecular Biology and Clinical Investigation	Regidor et al. [43]	Original article	2018	The second part of the trial aimed to investigate the oocyte quality, the ratio between follicles and retrieved oocytes, fertilization rate, and embryo quality in PCOS women undergoing IVF treatment	29 patients with PCOS	The placebo group showed a higher number of retrieved oocytes. MI and folic acid group (control) showed a better follicle/retrieved oocyte ratio, more metaphase II oocytes, and more grade 1 quality of the embryo. The control group also showed a nonsignificant increase in fertilization rate
4	European Review for Medical and Pharmacological Sciences	Unfer et al. [44]	Original article	2011	To compare the effect of MI and DCI on oocyte quality in euglycemic PCOS women		The study showed that there was no difference in the number of oocytes collected in two groups. However, the number of immature oocytes was less in the MI group along with an increase in the number of mature (metaphase II) oocyte, compared to DCI. Moreover, the number of top quality embryo and pregnancy rate was also higher in the MI group
5	International Journal of Endocrinology	Unfer et al. [45]	Review article	2016	To investigate the role of MIl and DCI in physiological involvement in PCOS and potential therapeutic use with assisted reproductive technologies		Inositol may have a role in improving hormonal and reproductive disturbance in PCOS women. It may also have a role in oocyte follicular development and oocyte maturation
6	Fertility and Sterility	Papaleo et al. [11]	Original article	2009	To determine the effects of oocyte quality in PCOS women undergoing ICSI	60 patients with PCOS	Total gonadotrophin required for stimulation was less in the MI group. However, the number of oocytes retrieved was not significantly different between the two groups. The mean number of degenerated vesicles and degenerated oocyte was less in the inositol group with the trend toward an increase in metaphase II oocytes
7	Reproductive BioMedicine Online	Mendoza et al. [46]	Review article	2017	To assess the effectiveness of MI and MI in improving oocyte or embryo quality and pregnancy rates for women with PCOS undergoing ICSI		MI was insufficient to improve the oocyte quality and embryo quality
8	Archives of Gynecology and Obstetrics	Laganà et al. [47]	Review article	2018	To evaluate whether oral MI supplementation is able to reduce the amount of gonadotropins and the length of controlled ovarian hyperstimulation in both PCOS and non-PCOS women undergoing IVF		In MI vs no intervention group, there was no difference in the number of oocytes collected and mature oocytes. In MI vs DCI, the number of mature oocytes was significantly higher in the MI group; however, there was no difference in total oocyte retrieved between the two groups
9	Medicine	Zheng et al. [48]	Review article	2017	To find out the effectiveness of inositol in IVF-ET, ovulation induction in infertile women		As a secondary outcome, the MI group had more number of grade 1 oocyte and less number of the germinal vesicles and degenerated oocytes. There was no difference in the number of oocytes collected between the two groups

As substantiated by the above data, the role of MI and DCI supplementation in improving oocyte and embryo quality in women with PCOS undergoing IVF has been investigated in the past in various studies. We aimed to review the association between inositol and its effect on oocyte or/and embryo quality in PCOS women who had undergone IVF and found some interesting results. The studies mentioned in Table 1 have been reviewed extensively, and we aimed to focus on oocyte and embryo quality results. Only IVF and intracytoplasmic injection (ICSI) cycles have been reviewed as our objective was to find out the effect of inositol complex on oocyte or embryo quality in IVF procedures. The number of metaphase II oocytes was taken as the marker for oocyte quality, and embryo quality was assessed based on the number of morphologically grade-one embryos.

The study by Lesione et al. was a prospective randomized study, and it aimed to find out the effect of MI and folic acid versus folic acid-only on oocyte quality, fertilization rate, embryo quality, and the ratio between follicles and retrieved oocytes in PCOS women undergoing IVF treatment [41]. This study revealed that a higher number of metaphase II and I are retrieved in MI and folic acid (MI + FA) group compared to the folic acid-only group. However, the results were not statistically significant. Regarding the number of good quality (grade one) embryo, there was a statistically significant (p<0.05) result in MI + FA group. Similarly, the fertilization rate was also significant in the MI + FA group. It was also noted that the number of oocytes retrieved was higher in the placebo (FA) group, and the ratio of follicle/retrieved oocyte was also lower in the test (MI + FA) group (p<0.05). The study concluded that MI supplementation resulted in a higher fertilization rate and, more importantly, a higher number of top-quality embryos and, therefore, has an overall effect on the quality of oocyte [41].

The study by Ciotta et al. showed that the number of oocytes retrieved was significantly higher in the MI group, and the number of immature oocytes (degenerated oocytes and germinal vesicles) was lower. However, there was no statistical significance in the number of metaphase II oocytes (though the trend was on the higher side) and fertilization rate. This study also revealed that the mean number of grade-one embryos available for transfer was also high in the inositol group (p<0.01). The authors concluded that MI has a role in oocyte maturation due to its insulin-sensitizing property [42].

It was demonstrated by Regidor et al. in their study that the ratio of follicle/retrieved oocyte was lower in the MI group (p<0.05); the fertilization rate was also statistically significant in the MI group [43]. Regarding the number of metaphase II oocyte, the trend favored the MI group, although it was not statistically significant, and the number of grade-one embryos was higher in the inositol group. This result was similar to the findings of Lesione et al. [41]. The authors suggested that MI has a positive role in maintaining the quality of the oocyte pool and increasing the fertilization rate [43].

Furthermore, Unfer et al. compared the effect of DCI and MI on oocyte quality in euglycemic PCOS women. They concluded that the number of mature oocytes and good-quality embryos was higher in the MI group compared to the DCI group [44]. These results support the hypothesis of the ovarian paradox effect of DCI [39]. Unfer et al., in another review, speculated that the inositol complex (MI and DCI) is a safe and effective treatment to increase follicular development and oocyte maturation [45]. However, the findings regarding the number of metaphase II oocyte and grade one embryo were not statistically significant.

In a study involving 60 women with PCOS, Papaleo et al. evaluated the effect of MI on the quality of oocyte in women undergoing ICSI. There was no difference in the number of oocytes retrieved between the two groups; however, there was a significant reduction in the number of degenerated oocytes and germinal vesicles, with a trend towards the increased percentage of metaphase II oocytes [11].

Conversely, two recent systematic reviews and metanalysis showed some different results. The review study by Mendoza et al. on the effect of inositol on women with PCOS undergoing ICSI argued that there is a lack of evidence to support the role of inositol complex in improving oocyte and embryo quality [46]. However, there was significant heterogeneity, and a small number of randomized control trials were included in the review. Another systematic review by Lagana et al. mentioned that MI improves the amount of gonadotrophin usage in the IVF cycle, although it shows no improvement in the total number of oocyte or mature oocytes. The improvement in the usage of gonadotrophins can be explained by the theory that inositol has a role in FSH signaling. However, there was heterogeneity among the studies included, and the quality of embryo was not assessed in all the studies [47]. Interestingly, another review by Zheng et al. stated that MI has a role in improving the number of good-quality embryos and reducing the number of germinal vesicles and degenerated oocytes. However, both PCOS and non-PCOS trials were included in their study [48].

## Conclusions

In this article, after reviewing the relevant literature, we can conclude that MI, an insulin-sensitizing agent, has a role in improving the quality of embryos in women with PCOS who undergo various ART procedures. However, the evidence is still not very clear for us to unequivocally recommend it for the improvement of oocyte and embryo quality. The quality of oocyte and, therefore, the embryo is one of the main rate-limiting and stressful factors associated with IVF success in PCOS cases. A large, multicentric, randomized controlled trial is therefore required before we can categorically accept or refute the role of MI in the betterment of oocyte and embryo quality in women with PCOS.
